# Nutritional Strategies to Optimize Performance and Recovery in Rowing Athletes

**DOI:** 10.3390/nu12061685

**Published:** 2020-06-05

**Authors:** Jooyoung Kim, Eun-Kyung Kim

**Affiliations:** 1 Office of Academic Affairs, Konkuk University, Chungju-si 27478, Korea; jykim1125@kku.ac.kr; 2 Division of Food Bioscience, College of Biomedical and Health Sciences, Konkuk University, Chungju-si 27478, Korea

**Keywords:** nutrition, performance, recovery, rowing, supplement

## Abstract

Rowing is a high-intensity sport requiring a high level of aerobic and anaerobic capacity. Although good nutrition is essential for successful performance in a rowing competition, its significance is not sufficiently established. This review aimed to provide nutritional strategies to optimize performance and recovery in rowing athletes based on a literature review. Following the guidelines given in the Preferred Reporting Items for Systematic Review and Meta-Analyses (PRISMA), we performed web searches using online databases (Pubmed, Web of Science, Wiley Online Library, ACS Publications, and SciFinder). Typically, a rowing competition involves a 6–8-min high-intensity exercise on a 2000-m course. The energy required for the exercise is supplied by muscle-stored glycogens, which are derived from carbohydrates. Therefore, rowing athletes can plan their carbohydrate consumption based on the intensity, duration, and type of training they undergo. For effective and safe performance enhancement, rowing athletes can take supplements such as β-alanine, caffeine, β-hydroxy-β-methylbutyric acid (HMB), and beetroot juice (nitrate). An athlete may consume carbohydrate-rich foods or use a carbohydrate mouth rinse. Recovery nutrition is also very important to minimize the risk of injury or unexplained underperformance syndrome (UUPS) from overuse. It must take into account refueling (carbohydrate), rehydration (fluid), and repair (protein). As lightweight rowing athletes often attempt acute weight loss by limiting food and fluid intake to qualify for a competition, they require personalized nutritional strategies and plans based on factors such as their goals and environment. Training and competition performance can be maximized by including nutritional strategies in training plans.

## 1. Introduction

Rowing is a sport in which an individual or group of people propels a boat on water using oars. In a rowing competition, the rank is decided based on the order of finishing a 2000-m course. Competitions can be classified according to the number of occupants in a boat (single, double/pair, four/quad, eight), the presence of a coxswain steering, and whether the boat is sculled (two oars per person) or rowed (one oar each) [1]. In general, a rowing competition takes place on a 2000-m course and the exercise lasts 6–8 min, with a number of muscle groups being involved (legs about 65%, back about 25%, and arms about 10%) [2]. During 2000-m rowing, muscle glycogen is mainly used as the energy substrate of the aerobic energy system, accounting for about 77% of the total energy supply, while 33% of the total energy supply is supplied from the anaerobic energy system (phosphates, ATP, and phosphocreatine), which contributes to the energy supply in the initial stage of rowing [2]. In order for rowing athletes to improve their records and win medals, highly developed aerobic and anaerobic systems are required [3]. Hence, rowing athletes train to improve not only aerobic and anaerobic capacities, but lactate tolerance, strength, and power as well [4].

Such training must be supported by sufficient nutrition. Not only does nutrition improve performance by optimizing training adaptation of an athlete, but it is also very important to keep an athlete healthy [5]. Boegman and Dziedzic [4] claim that an individualized and flexible nutrition plan is essential in order to fulfill the daily, weekly, and cyclic nutritional requirements of rowing athletes. Recently, Lewis et al. [6] applied a four-month nutrition intervention to rowing athletes with unexplained underperformance syndrome (UUPS; also known as overtraining syndrome) and showed evidence of performance enhancement, with a specific achievement of becoming European champions. Kurgan et al. [7] reported that the bone mineral density and bone mineral content in female heavyweight rowing athletes with suitable energy availability were stably maintained over a season. However, investigations of the nutrition for rowing athletes are still limited, and there are few nutritional guidelines for before, during, and after of the training or competition to enhance and maintain performance. Although Boegman and Dziedzic [4] presented nutritional strategies for elite open-weight rowing athletes, they mainly focused on before and during nutrition, and there was no information on the recommended kind of carbohydrates, total calorie intake, and after nutrition. In addition, the nutritional information for lightweight rowing athletes was also insufficient.

Doering et al. [8] found that, while athletes are aware of the importance of nutrition, they lack significant knowledge about carbohydrate and protein intake. Heikkilä et al. [9] also found that athletes and coaches often lack knowledge on nutrition. They suggested that athletes need to obtain sufficient nutrition knowledge so as to understand the importance of food choices for competitions, recovery, and overall health. These studies [8,9] imply the need to provide a practical guideline for both athletes and their training coaches. Therefore, the aim of this study was to carry out a literature review of published experimental research on rowing athletes and provide scientific evidence-based nutrition strategies to optimize the performance and recovery of rowing athletes.

## 2. Materials and Methods

This review was conducted based on the guidelines presented in Preferred Reporting Items for Systematic Review and Meta-Analyses (PRISMA). In this review, online databases such as Pubmed, Web of Science, Wiley Online Library, ACS Publications, and SciFinder were used to collect and analyze nutrition-related articles that could boost performance and recovery of rowing athletes. Keywords used in the search included “rowing”, “rower”, “nutrition”, “carbohydrate”, “protein”, “hydration”, “performance”, “recovery”, and “supplements”, and these words were sometimes used in combination. The combination of the key words included “rowing and nutrition”, “rowing and carbohydrate”, “rowing and protein”, “rowing and hydration”, “rowing and supplements”, “rower and nutrition”, “rower and carbohydrate”, “rower and protein”, “rower and hydration”, and “rower and supplements”.

The literature search was conducted by two researchers (J.K. and E.-K.K.) independently using titles, abstracts or both, and when there was a disagreement between them, it was resolved through subsequent consensus meetings. The inclusion and exclusion criteria for literature review were established by the two researchers (J.K. and E.-K.K.). As for the inclusion criteria, this review included: (1) Full reports (original articles, literature reviews, and case reports) published in peer-reviewed journals, (2) studies written in English, (3) studies involving rowing athletes as subjects, and (4) studies that measured indicators that can determine the performance and recovery of rowing athletes. On the other hand, the retrieved article titles were checked first, and all irrelevant articles were excluded. Then, the researchers looked through the abstract and body of the articles, and all those not related to performance or recovery nutrition were excluded. As for the selected literature, eligibility was finally determined after the two researchers (J.K. and E.-K.K.) read the entire literature.

**Figure 1:**
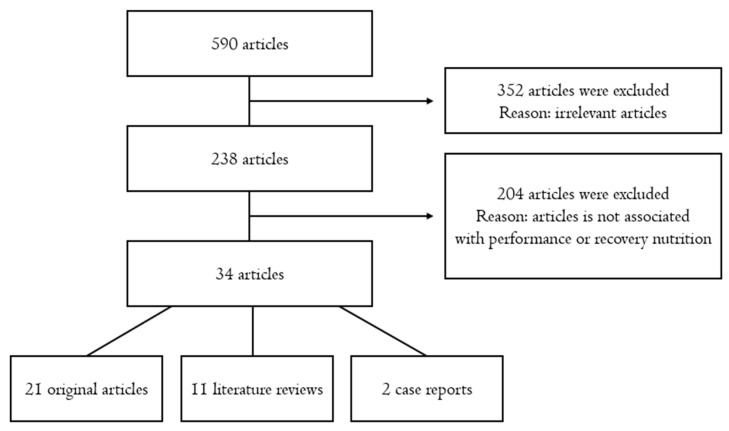
Flowchart of the literature search process.

Through this process, a total of 34 articles were finally selected and reviewed in this study, including 21 original articles, 11 literature reviews, and 2 case reports. The flowchart of literature search process is presented in Figure 1.

## 3. Nutrition for Rowing Performance in Competition or Training

Rowing is a high-intensity sport that involves both the upper and lower body. As described in the previous section, a rowing competition takes 6–8 min on a 2000-m course and requires a sufficient amount of glycogen in muscles for energy [2,10]. Glycogen can be stored in muscle and in the liver through carbohydrate consumption. Whereas it is a basic fuel for rowing performance, glycogen also plays a role in helping prevent fatigue during a competition or training, as well as for cognitive function enhancement and maintenance [11]. Glycogen is not only an energy substrate but is also considered a regulator in signaling pathways that regulate exercise-induced adaptions [12]. If a rowing athlete neglects carbohydrate intake and participates in a competition with a low level of glycogen, the athlete could experience fatigue early in the competition, thus leading to a reduced rowing performance.

### 3.1. Before Training or Competition

Since rowing athletes train from 1–3 times a day and 5–6 times a week and the intensity of training is high, a sufficient amount of carbohydrates must be consumed prior to training in order to increase glycogen availability [13,14]. In fact, a group of athletes on a high-carbohydrate diet (10 g/kg/day) had more glycogen stored in muscle than a group of athletes on a moderate-carbohydrate diet (5 g/kg/day) and showed a higher level of enhancement in power output during a rowing training [13]. Some athletes skip breakfast and still participate in morning or afternoon trainings, which is an eating habit that can have negative effects on training performance. In a recent study, rowing athletes who skipped their carbohydrate-rich breakfast showed delays in their 2000-m time trial records measured in an afternoon training and reported high ratings of perceived exertion (RPE), which represents subjective training intensity [14].

The average calorie intake of rowing athletes is in the range of 2600 kcal to 4900 kcal. Depending on the training, it can reach up to approximately 7000 kcal (Table 1). Out of the total calorie intake, carbohydrate make up approximately 4.6–6.3 g/kg [4]. It is recommended that rowing athletes establish an individualized plan for their carbohydrate intake based on the intensity, duration, and type of training. For example, 3–5 g/kg/day of carbohydrate is recommended for a low-intensity training or a skill-based activity; 6–10 g/kg/day for a 1–3 h training with moderate–high intensity; and 8–12 g/kg/day for a 4–5 h training with moderate–high intensity [15]. If an athlete did not have a sufficient intake of carbohydrates in previous meals, an additional carbohydrate intake can be done 30–60 min before training (Table 1). In the past, an intake of carbohydrates 30–60 min before training was said to cause rebound or reactive hypoglycemia during training. However, a study reported that pre-training carbohydrate intake does not have a negative effect on performance [16].

### 3.2. During Training or Competition

Carbohydrate supplements for performance maintenance taken during training or competition must be easily digested and absorbed because the gastrointestinal (GI) tract plays an important role in transporting carbohydrates and fluid during training or competition [17]. However, not all types of carbohydrate are allowed. Athletes may intake carbohydrates such as glucose, sucrose, maltose, and maltodextrin [12]. In general, sports drinks and gel or low-fat, low-protein, and low-fiber solid bars are taken as supplements (Table 1). Sometimes, rowing athletes can use a carbohydrate mouth rinse during training [18]. As the term itself suggests, a carbohydrate mouth rinse is a method in which carbohydrates (sports drink or maltodextrin) are taken orally and used to rinse the mouth. It is regarded as an effective carbohydrate intake strategy during training [19]. In fact, Jensen et al. [19] let rowing athletes perform maximal isometric knee extensions. Then, athletes rinsed their mouths with 8% maltodextrin, repeated the same muscle contraction, and found that their performance improved due to diminished fatigue. Although the mechanisms that explain such changes have not been clearly identified, a theory has been put forward: Carbohydrate intake stimulates taste receptor cells distributed in the mouth, and taste cues then generate and transmit electrical activity to gustatory neurons, which leads to performance enhancement [20]. To perform a mouth rinse during training or competition using carbohydrates, the athlete must rinse their mouth with a sports drink or maltodextrin and spit it out after 5–10 s. Mouth rinses can be performed at 10–15 min intervals [18]. This method can be effective for a high-intensity training with a duration of less than one hour. A carbohydrate mouth rinse can be particularly helpful for rowing athletes who frequently experience gastrointestinal issues caused by drinks consumed during training.

### 3.3. Use of Performance Enhancing Supplements

Many athletes take various scientifically proven supplements in order to sustain high-intensity training, improve their records, and win competitions. Rowing athletes also take supplements for purposes such as enhancing performance (Table 2). Typical performance-enhancing supplements used by rowing athletes include β-alanine, among others [21]. Recently, Turnes et al. [21] claimed that the use of β-alanine is a popular and effective preconditioning strategy used to enhance performance among rowing athletes. It is known for its ability to maintain or enhance performance by increasing carnosine concentration in muscle to maintain muscle pH and delay exercise-induced fatigue [22]. In addition, since carnosine acts as a neurotransmitter, it improves response times and accuracy [23]. Several studies have investigated the supplementary effects of β-alanine on rowing athletes: Baguet et al. [23] found that muscle carnosine content significantly increased after taking 5 g of β-alanine supplement every day for seven weeks and showed a positive correlation with 2000-m performance enhancement, and Hobson et al. [24] showed that chronic supplementation of β-alanine (6.4 g per day for four weeks) in well-trained rowing athletes enhanced their 2000-m rowing performance. Rowing athletes can take β-alanine with meals, the typical side effect of which is minimizing paresthesia that subsides upon dividing the dose over time or stopping intake [25].

Caffeine is a naturally occurring stimulant found in numerous plants. Thus, it is widely used as a supplement to increase strength and endurance and the glycogen sparing effect [26]. According to a study by Del Coso et al. [27] that investigated caffeine usage among athletes, rowing athletes were reported as one of the groups with the highest caffeine consumption. Several studies supported the performance enhancement effect of caffeine supplements in rowing athletes: Anderson et al. [28] divided oarswomen into two groups who differed in the amount of caffeine intake prior to rowing (6 mg/kg or 9 mg/kg) and showed that the performance of the 9 mg/kg intake group was enhanced; Bruce et al. [29] reported that both 6 mg/kg and 9 mg/kg reduced performance time and increased mean power; and Scott et al. [30] showed that taking caffeinated carbohydrate gel (21.6 g carbohydrate, 100 mg caffeine) 10 min before rowing reduced 2000-m rowing time. Results regarding the effect of caffeine intake on rowing performance are consistent: In their meta-analysis, Grgic et al. [31] suggested that caffeine effectively increases rowing ergometer performance, and another meta-analysis by Turnes et al. [32] reported that 6 mg/kg caffeine intake has the highest possibility of enhancing performance.

Sodium bicarbonate is often recommended for performance enhancement of rowing athletes as well. It has similar functions to β-alanine (the ability to buffer H +) [33]. Hobson et al. [34] reported intake of 0.3 g/kg sodium bicarbonate enhanced the athletic performance. However, several studies showed that sodium bicarbonate supplement has no effect on rowing performance enhancement [35,36] and that caffeine supplementation is more effective for performance enhancement [37,38]. Upon comprehensive review of various studies, there is no sufficient evidence to strongly encourage rowing athletes to take sodium bicarbonate supplements.

A metabolite of leucine, β-hydroxy-β-methylbutyric acid (HMB), has recently received attention in the field of sports nutrition [39]. HMB is shown to benefit athletes by increasing muscle mass and improving muscle recovery. However, in a study on rowing athletes that investigated variables related to endurance and body composition [40], Durkalec-Michalski and Jeszka showed that supplementing rowing athletes with a daily dose of 3 g of HMB for 12 weeks led to an increase in aerobic capacity and peak anaerobic power, and decrease in fat mass. Also, they suggested tentative mechanisms, such as mitochondrial biogenesis due to AMP-activated protein kinase (AMPK), Sirt 1 activation, and improved efficiency of carbohydrate, glycogen, and fat metabolisms, to explain the observed changes. A recent study [41] reported that supplementing rowing athletes with a combination of HMB (3 g/day) and creatine (0.04 g/day) for 10 weeks did not change muscle mass but significantly improved aerobic power. The authors suspected that the improvement in aerobic power was a result of a synergy effect between HMB that caused fiber transformation (driving fast-to-slow fiber switch) together with increased gene expression of gamma co-activator 1-alpha (PGC-1α) and creatine that increased energy availability during exercise via elevated phosphocreatine level in muscle [41]. This means that rowing athletes can save glycogen and gain energy via oxidative capacity when they need endurance. These results imply that HMB has a potential function to promote enhancement of variables related to aerobic capacity. Whereas there is a need for further studies on its combinatory supplementation with creatine, our review on current literature suggests that we can give HMB supplementation a shot with the purpose of enhancing the performance of rowing athletes in training or competition.

Meanwhile, several studies reported that beetroot juice, rich in nitrate (NO3-), can be helpful for rowing athletes [42,43]. Nitrate has a basic mechanism for vasodilation, in which it promotes the synthesis of nitric oxide (NO), and induces effects such as increased blood flow, promotion of ATP synthesis in mitochondria, and decreased ATP cost in muscle force production [44]. Also, beetroot juice has been introduced as a supplement with strong scientific evidence in the IOC consensus statement and the Australian Institute of Sport (AIS) and International Society of Sports Nutrition (ISSN) [45]. Bond et al. [42] showed that performance time improved in the later stages of rowing performance when well-trained junior male rowing athletes consumed beetroot juice that contained 5.5 mmol nitrate every morning (250 mL) and afternoon (250 mL) for six days. Hoon et al. [43] also observed that 2000-m rowing performance improved when highly trained male rowing athletes consumed 140 mL beetroot juice that contained 8.4 mmol of nitrate two hours prior to exercise. Maughan et al. [45] showed that 5–9 mmol (310–560 mg) of nitrate-rich beetroot juice had an effect on acute performance 2–3 h after its intake and that intake for more than three days may also be beneficial. These results suggest that nitrate-rich beetroot juice can be considered as an effective supplement for performance enhancement in rowing athletes.

## 4. Nutrition for Recovery after Rowing Competition or Training

Recovery is a popular topic among rowing athletes because they experience physiological phenomena such as glycogen decrease and dehydration after trainings or competitions [15]. Also, after such exercises, various inflammatory markers increase, promoting muscle protein breakdown and causing muscle damage and soreness. If inflammation persists, it can affect immune, metabolic, and psychological functions [46]. Hence, neglecting recovery can lead to a decline in the overall condition of the body and increase the risk of injury or UUPS from overuse [47]. For the recovery nutrition of a rowing athlete, refueling, rehydration, and repair must be considered [48].

### 4.1. Refueling

For refueling after training, carbohydrate intake is necessary (Table 3). Since the average glycogen replenishment in muscle is generally about 5-6 mmol per hour, recovery after a high level of depletion takes about 24 h [11]. Hence, carbohydrate intake after a high-intensity training must be done as soon possible. The high level of glycogen synthase and insulin sensitivity right after training facilitate a faster and increased storage of glycogen [49]. This is particularly important when the recovery period is short due to consecutive rowing training sessions (e.g., training throughout a day such as morning, afternoon, and evening sessions). Carbohydrate products with a high glycemic index (GI) can give an advantage in glycogen storage [50]. Burke et al. [51] found that carbohydrate products with a moderate or high GI resulted in increased glycogen storage during the recovery period compared to those with a low GI. Also, it has been reported that carbohydrate products can be taken in the liquid or solid form or as a meal or a snack [52,53]. The recommended carbohydrate is about 1.2 g/kg [4]. For instance, a rowing athlete who weighs 60 kg needs an intake of 72 g of carbohydrates right after a training. By focusing on glycogen replenishment through the planning of the form, type and amount of carbohydrate intake during the recovery period, and based on the intensity and duration of the training, athletes can prepare for the next training or competition while full of energy.

### 4.2. Rehydration

Rowing athletes also need to pay attention to rehydration during the recovery period, as it is the area of nutrition that is most easily neglected during recovery (Table 3). Fluid must be consumed right after training. However, rather than drinking a very large amount within a short period of time, multiple intakes spread across the recovery time are better [15]. In general, weight loss after training reflects a loss of fluid. Monitoring weight loss is the easiest and best method to measure hydration status [54]. After a 2000-m rowing competition, an athlete experiences a weight loss of approximately 1.5–2 kg. The amount of fluid intake must be 1.5-times the amount of weight loss after a competition or training [55]. The recommended multiplication factor used to be 1, but taking into account the fluid loss from urination and other activities after exercise, an intake of 1.5-times the amount of weight loss is effective in improving and maintaining hydration status. If there has been a marked weight loss from sweating after a competition or training, a rowing athlete can drink commercially available sports drinks or eat food with sodium (Na^+^) and water [56]. Based on the environmental conditions during trainings or competitions, a rowing athlete should be able to decide on the type of fluid to intake.

### 4.3. Repair

Repair is related to protein intake, which is definitely necessary for protein synthesis (Table 3). According to scientific evidence, the amount and type of intake and the interval between intakes affect recovery after training [57,58]. The recommended amount of protein intake in the early stage of recovery is about 20–25 g, which can increase up to 40 g based on factors such as age and muscle mass [58]. The amount of protein intake can be converted into a relative value. For recovery, 0.3–0.4 g/kg protein can be consumed immediately after a training or competition [59]. The most important considerations in the selection of protein food for recovery include whether it can be easily digested and absorbed and how much essential amino acids and leucine it contains [60]. A rowing athlete can consume various foods with essential amino acids. Further, if intake of protein through diet is difficult, an athlete may consume whey protein, which is the protein fraction of the byproducts of cheese production using milk. Due to its fast absorption rate, whey protein is already consumed as a source of protein among many athletes [60]. It can be easily absorbed and digested and has a rich essential amino acid content, especially leucine, which activates a regulator of protein synthesis called ‘mammalian target of rapamycin’ (mTOR) in muscle to help recovery [61]. In general, 20–25 g protein (as part of a meal or snack) should be consumed every 3–5 h for recovery [62]. In order to maximize protein synthesis, the intake of casein protein before sleep can be recommended [63,64]. Casein protein comprises 80% of the proteins in milk and is considered in pre-sleep nutritional strategies because, due to its slow digestion and absorption rates, it can constantly supply amino acids in the body [58]. Therefore, an intake of 40 g casein protein before 30 min prior to sleep, a period lacking in nutritional supply, can bring a number of advantages for recovery [65].

## 5. Nutrition for Recovery after Acute Weight Loss in Lightweight Rowing Athletes

This section is applicable to lightweight rowing athletes who are subject to the weight limit for participation in competitions. On average, the weight limit is 72.5 kg for males and 59 kg for females. Therefore, these athletes often attempt acute weight loss by increasing training volume or limiting food and fluid intake within a short period of time before a competition [66,67]. The International Federation of Rowing Association (FISA) restricts athletes from taking radical measures to lose weight and recommends that any weight loss performed 24 h prior to a competition should not exceed 1 kg (Table 4). However, despite such recommendations, lightweight rowing athletes attempt to lose more weight. A study [68] reported that lightweight rowing athletes that had undergone heavy weight loss showed a decline in performance. In another study [66], the actual Na^+^ and fluid intake was lower than the recommended amount among lightweight rowing athletes despite their awareness of the importance of recovery after acute weight loss.

Slater et al. [69] showed that while the plasma volume of lightweight rowing athletes who had undergone approximately 4% body mass loss and aggressive nutritional recovery strategies (carbohydrate: 2.3 g/kg; Na^+^: 34 mg/kg; and fluid: 28.4 mL/kg) two hours prior to a competition was significantly decreased, the strategies had no significant effect on their 1800-m time-trial performance. On the other hand, in another study by Slater et al. [70], rowing athletes still showed a decline in their 2000-m time-trial performance despite following the nutrition intake plan for recovery (carbohydrate, Na^+^, and fluid) after approximately 4% body mass loss. We think that these contradicting results are due to differences in the environmental conditions and distance under which the rowing performance was measured: Slater et al. [69] measured performance from a 1800-m course under cool conditions, whereas Slater et al. [70] measured performance from a 2000-m course under hot conditions.

Whereas we must still be careful in interpreting and applying the contrasting results of previous studies, we think that lightweight rowing athletes who have attempted acute weight loss need to actively engage in nutrition intake for recovery despite being given a short period of time before a competition. Although it takes a long time to completely replenish the glycogen and fluid depleted from weight loss, a decline in rowing performance during a competition can be minimized by actively taking nutrition until the competition. In particular, an intake of fluid or a combination of water and carbohydrate/Na^+^ is recommended (Table 4). Slater et al. [71] compared three groups of lightweight rowing athletes who had undergone acute weight loss (about 5.2%) and in whom nutrition contents differed (fluid, carbohydrate/Na^+^, and combination of water and carbohydrate/Na^+^) and reported that there was a significant decline in performance in the carbohydrate/Na^+^ intake group only.

## 6. Conclusions

In the Olympic games, a fierce competition within a short period of time, a change of about 1% in the average speed of rowing is a potential factor that can result in winning a medal [4,38]. The effort that rowing athletes put in for the 1% change will pay off only when they follow both the training that takes into account the physiological characteristics of competition and the nutrition strategy that is optimized for them. Although further studies on rowing athletes are required, based on our literature review, it is clear that a nutritional approach and application have positive effects on performance and recovery. Therefore, rowing athletes should establish appropriate nutritional strategies based on factors such as their goals and environment. Also, coaches who train rowing athletes should establish a yearly or monthly training plan that includes appropriate nutritional strategies for each cycle in order to maximize training effects and in-game performance.

## Figures and Tables

**Table 1:** Nutrition points for the performance of rowing athletes.

Periods	Nutrition Points
Before training or competition	In rowing training or competition, both anaerobic and aerobic metabolism are used, and glycogen is utilized as a very important energy substrate. In general, the average calorie intake of a rowing athlete is between 2600 kcal and 4900 kcal (possible intake up to 7000 kcal). - Carbohydrate intake is about 4.6–6.3 g/kg. Carbohydrate intake can be varied depending on the intensity, duration, and type of training. - Low-intensity training or a skill-based activity: 3–5 g/kg/day - 1–3 h training with moderate–high intensity: 6–10 g/kg/day - 4–5 h training with moderate–high intensity: 8–12 g/kg/day In case of insufficient carbohydrate intake at regular meals, it is possible to replenish easily digestible carbohydrates 30-60 min before training or competition.
During training or competition	Rowing athletes can consume carbohydrates in various forms such as glucose, sucrose, maltose, and maltodextrin. In general, sports drinks and gel or low-fat, low-protein, and low-fiber solid bars can be consumed for supplementation. Sometimes, carbohydrate mouth rinse can be applied. - This method can be effective in high-intensity training performed within 1 h. - After taking a sip of sports drink or maltodextrin, rinse in the mouth for 5–10 s and spit it out again. - In this way, carbohydrate mouth rinse is performed every 10–15 min.

**Table 2:** Effect of performance enhancing supplements on rowing athletes.

Supplements	Reference	Subjects	Trials	Main Findings
β-alanine	Baguet et al. [23]	Elite male and female rowers (*n* = 19)	5 g/day for 7 weeks (divided over 5 doses of 1 g, ingestion with 2 hr intervals)	Rowing speed ↑
	Hobson et al. [24]	Well-trained male rowers (*n* = 20)	6.4 g/day for 4 weeks (2 × 800 mg/time, 4 times/day separated by 3–4 hr)	2000-m rowing performance ↑
Caffeine	Anderson et al. [28]	Competitive male rowers (*n* = 8)	6 or 9 mg/kg (timing: 60 min before exercise)	Rowing performance time↑ (both doses of caffeine had a similar effect) Mean power output ↑ (9 > 6 mg/kg) First 500 m time of 2000-m row ↑ (9 > 6 mg/kg)
	Bruce et al. [29]	Well-trained male rowers (*n* = 8)	6 or 9 mg/kg (timing: 60 min before exercise)	Rowing performance time↑ (both doses of caffeine had a similar effect) Mean power output ↑ (both doses of caffeine had a similar effect)
	Scott et al. [30]	University sports performers (*n* = 13)	21.6 g of CHO and 100 mg of caffeine (caffeinated CHO gel, timing: 10 min before exercise)	2000-m rowing performance ↑
	Carr et al. [37]	Well-trained male and female rowers (*n* = 8)	6 mg/kg (timing: 30 min before exercise)	2000-m mean power ↑
	Christensen et al. [38]	Elite male rowers (*n* = 12)	3 mg/kg (timing: 45 min before exercise)	Total distance ↑ Mean power ↑
Sodium bicarbonate	Hobson et al. [34]	Well-trained male rowers (*n* = 20)	0.3 g/kg (timing: 120 min before exercise)	2000-m rowing performance ↑
	Driller et al. [35]	National male rowers (*n* = 12)	0.3 g/kg for 4 weeks (timing: 90 min before exercise)	2000-m power = 2000-m time =peak power output = power at 4 mmol/L lactate threshold =
	Kupcis et al. [36]	Elite male rowers (*n* = 7)	0.3 g/kg (timing: 70–90 min before exercise)	Rowing performance time =
	Carr et al. [37]	Well-trained male and female rowers (*n* = 8)	Acute: 0.3 g/kg (timing: 120 min before exercise) Chronic: 0.5 g/kg for 3 days (ingestion with meals and snacks)	2000-m rowing performance = (both acute and chronic)
	Christensen et al. [38]	Elite male rowers (*n* = 12)	0.3 g/kg (timing: 75 min before exercise)	Total distance = Mean power =
HMB	Durkalec-Michalski and Jeszka [40]	Elite male rowers (*n* = 16)	3x1 g/day for 12 weeks (timing of 3 doses: Upon waking, immediately after training, and before sleep)	VO2 max ↑Ventilatory threshold ↑Peak anaerobic power ↑
	Fernández-Landa et al. [41]	Elite male rowers (*n* = 28)	3 g/day for 10 weeks (+ creatine: 0.04 g/kg and chocolate shake: 1 g/kg of CHO and 0.3 g/kg protein; timing of training day: In the half hour after training, and timing of off day: 30 min before sleep)	Aerobic power ↑
Beetroot juice	Bond et al. [42]	Well-trained junior male rowers (*n* = 14)	5.5 mmol nitrate/day for 6 days (timing: Every morning and afternoon)	Maximal rowing-ergometer repetitions ↑
	Hoon et al. [43]	Well-trained male rowers (*n* = 10)	8.4 or 4.2 mmol nitrate/day (timing: 2 hr before exercise)	2000-m rowing performance ↑ (8.4 > 4.2 mmol nitrate)

HMB, β-hydroxy-β-methylbutyric acid; ’↑’: Significant improvement, ’=’: No significant difference.

**Table 3:** Nutrition points for the recovery of rowing athletes.

Components	Nutrition Points
Refueling	The most important goal is the carbohydrate intake for glycogen replenishment. Timing of carbohydrate intake: Immediately after the training or competition (the sooner the better). Type of carbohydrate intake: High glycemic index (GI) carbohydrate. Carbohydrate form: Liquid or solid form or as a meal or a snack. Amount of carbohydrate intake: 1.2g/kg.
Rehydration	The most important goal is ensuring sufficient fluid intake. Timing of fluid intake: Immediately after the training or competition. The weight loss after the training reflects the loss of fluid (Monitoring of weight change is required). Amount of fluid intake: 1.5-times the amount of weight loss. Sports drinks or food with sodium (Na+) and water can be consumed.
Repair	The most important goal is to facilitate muscle protein synthesis. Protein type: Whey protein (easy digestion and absorption, rich in essential amino acids and leucine). Amount of protein intake: From about 20–25 g to 40 g; relative value of the intake is about 0.3–0.4 g/kg. Protein intake distribution: Intake of every 3–5 h is recommended. To promote recovery, approximately 40 g of casein protein can be consumed 30 min before sleep.

**Table 4:** Nutrition points for recovery after acute weight loss of lightweight rowing athletes.

Nutrition Points
In case of acute weight loss performed at 24 h before the competition, the loss of weight should not exceed 1 kg. In case of acute weight loss, active nutrition intake for recovery is required, even if only a short time is given before the competition. For recovery after acute weight loss, the consumption of fluid or combination of water and carbohydrate/Na^+^ is recommended.
